# Government use licenses in Thailand: The power of evidence, civil movement and political leadership

**DOI:** 10.1186/1744-8603-7-32

**Published:** 2011-09-12

**Authors:** Suwit Wibulpolprasert, Vichai Chokevivat, Cecilia Oh, Inthira Yamabhai

**Affiliations:** 1 Ministry of Public Health, Nonthaburi, Thailand; 2 Government Pharmaceutical Organizations, Bangkok, Thailand; 3 Health Intervention and Technology Assessment Program, Nonthaburi, Thailand

## Abstract

This paper attempts to describe and analyse the policy processes that led to the granting and implementation of the government use licenses to enable the import and production of generic versions of medicines patented in Thailand. The decision to grant the series of government use licenses was taken despite much domestic and international controversy. The paper demonstrates that the policy processes leading to the granting of government use licenses are a successful application of the concept of  "the triangle that moves the mountain". This is a well-known conceptualisation of a philosophical and strategic approach to public policy advocacy in Thailand, which propounds that the effective bridging of three powers; a.) Knowledge and evidence generated by research and analysis, b.) Civil society movements and public support, and c.) Leadership of policy makers and politicians; in a synergistic "triangle" can move "mountains", meaning the resolution of seemingly insurmountable problems. The paper provides insights into the policy context for the decision and analyses the roles of key actors, their motivations and the policy processes in the country.

## Introduction

In 2001, Trade Ministers at the World Trade Organization (WTO) Ministerial Conference in Doha signed a declaration confirming their common understanding that the provisions of the Agreement on Trade-Related Aspects of Intellectual Property Rights (TRIPS) did not prevent governments from taking the necessary policy and legal measures to promote access to medicines and protect public health [1]. The Doha Declaration on the TRIPS Agreement and Public Health put an end to the long-running debate at the WTO that pitted developing countries against the developed countries on the issue of limitations on intellectual property rights to enable access to affordable medicines [2]. The Doha Declaration explicitly states that the TRIPS Agreement should be interpreted in a way that promotes access to medicines for all, and that countries were within their rights to take certain measures to limit intellectual property rights (collectively known as the "TRIPS flexibilities") when public health interests demand it [3].

Although the Doha Declaration was sought by the developing country governments to affirm their rights, there have only been a few cases of countries utilising TRIPS flexibilities since the adoption of the Declaration [4]. One reason for this is the persistent fear - particularly among smaller developing countries - that the use of TRIPS flexibilities will invite political pressure and trade sanctions from governments of the developed countries in which the patent holding pharmaceutical companies are based, seeking to protect the interests of those companies [3].

In 2006 and 2008, the government of Thailand decided to grant government use licenses^1 ^to enable the import and local production of the generic versions of seven medicines that are patent protected in Thailand. The first license, granted in November 2006, was for the Antiretroviral (ARV) drug, efavirenz (EFV) [5]. The second and third licenses were granted in January 2007, for the second-line ARV combination of lopinavir/ritonavir (LPV/r) [6] and clopidogrel (an antiplatelet agent used in the treatment of coronary artery disease) [7]. Four additional licenses were granted in January 2008 for cancer drugs, letrozole [8], docetaxel [9], erlotinib [10], and imatinib (which are used in the treatment of breast and lung cancers, gastrointestinal stromal tumor and leukaemia) [11]. The government use licenses for the seven medicines were granted under Section 51 of the Patent Act 1979 of Thailand, which authorizes the government use of patents in the general public interest, so that "any ministry, bureau or department of the Government" may exercise the right in any patent "to carry out any service for public consumption". Nevertheless, the government's action attracted controversy both within and beyond the country.

In Thailand, the granting of government use licenses had its share of critics who were concerned, among other things, that the political and economic costs of the licenses would outweigh the benefits. They feared the imposition of trade and other sanctions from foreign governments opposed to such a measure, as well as retaliation from the affected pharmaceutical companies [12,13]. The US government and European Commission, despite having signed the Doha Declaration, sought to exert political pressure on Thailand through various means, including the threat of trade sanctions. The political pressure exerted on Thailand by the US and the European Commission (EC) was a striking example of the discrepancy between political rhetoric at the international stage and policy practice at the bilateral level [14,15].

This paper describes and analyses the policy processes that, in the face of these pressures, led to the granting and implementation of the government use licenses to enable the import and production of generic versions of medicines patented in Thailand. The aim is to provide insights into the political context, the roles of key actors and their motivations, and the processes which led to these decisions being taken and to demonstrate that the decision to grant the government use licenses was a successful application of the concept of "the triangle that moves the mountain".

### "The triangle that moves the mountain"

**Figure 1:**
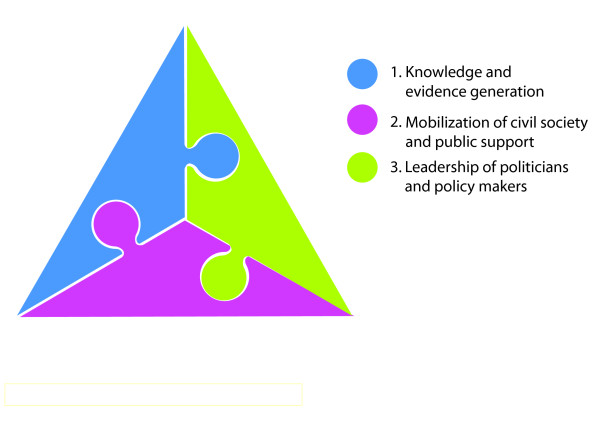
**Triangle that moves the mountain**.

This is a well-known conceptualisation of a philosophical and strategic approach to policy advocacy by Dr Prawase Wasi, a central figure in Thailand's political and social reform and public health arenas. Dr Wasi's triangular model (Figure 1) stresses the need for synergistic interactions between three powers; i) the power of wisdom, ii) social power, and iii) political power, to affect significant policy change or reform [16]. According to this approach, the effective bridging of three powers; a.) knowledge and evidence generated through research and analysis, b.) mobilization of civil society and public support, and c.) leadership of politicians and policy makers - in a "triangle" can result in the resolution of seemingly insurmountable problems. While each aspect on its own is not enough, when combined, the forces can move a mountain.

Dr Wasi synthesised this concept from processes which led to political reform in 1997 that culminated in the adoption of the 1997 Constitution, B.E. 2540 (1997), popularly known as the People's Constitution^2^[17]. Since then, this strategic approach to policy advocacy has been used in a number of contexts to successfully effect policy reform in Thailand, including the national health reform and the alcohol policy development process [18,19].

The remainder of the paper is organised as follows. The next section describes the historical aspects of the patent and access to medicines issue in Thailand, examining how key actors were sensitised on the interlinked issues of rising costs of ARVs, pharmaceutical patents and the use of the TRIPS flexibilities. Then, the paper examines the policy and decision-making processes within the government in the lead up to the decision to grant the government use licenses. This part comprises an analysis of the convergence of key actors and processes both in the domestic and international spheres, i.e. how the different actors in various processes came together as a triangle to support the grant and implementation of the government use licenses. The last section describes the different aspects of policy implementation by the relevant authorities, including the means by which opposition from within the government and the pharmaceutical industry was addressed.

### A Long March

It can be said that Thailand has had more than two decades of experience in addressing the debates on patents and access to medicines. In the mid-1980s, during the General Agreement on Tariffs and Trade (GATT) Uruguay Round of multilateral trade negotiations leading up to the establishment of the WTO and its Agreements (including the TRIPS Agreement), the US administration began to put pressure on developing countries, including Thailand [20], to increase their levels of intellectual property rights protection. These demands led to increased interest and research on the costs and benefits of intellectual property protection on medicines among Thai academics, public health personnel and people working for health-related non-government organizations (NGOs) led by the Drug Study Group^3 ^[21].

The Drug Study Group conducted a study on the impact of enhanced patent protection on medicines on Thailand's health care system which revealed that higher levels of patent protection would significantly increase the value of drug imports by 72 percent and hinder the development of the national drug industry [22]. These findings were cited and used by those who opposed the US bilateral pressures to amend the Thai Patent Act. The social movement against the US proposal, although not successful, was able to delay for six years the amendment of the Patent Act until 1992. The Act was eventually amended to extend the term of patent protection from 15 to 20 years and to provide additional patent protection for pharmaceutical products, in addition to the process patent protection already provided. This amendment however was still eight years ahead of the 2000 deadline for developing countries to implement the provisions of the TRIPS Agreement.

During the late 1990s, the global movement for equitable access to ARV treatment gained increasing momentum, fuelled by the public outcry against the high cost of patented ARV treatments [23]. Advocacy efforts to promote access to essential health services in Thailand, including access to medicines, was significant in the area of HIV/AIDS [24]. The first attempt to use TRIPS flexibilities in Thailand was the request for a compulsory licence for didanosine (DDI) in 1999 [25]. DDI is used in combination with other ARVs as part of the highly active antiretroviral therapy (HAART). At the time, the not-for-profit Government Pharmaceutical Organization (GPO), had initiated an effort to produce and supply the generic version of DDI [26]. The patent holder, Bristol-Myers Squibb (BMS), however, halted the production of GPO's DDI, alleging GPO's tablet formulation of DDI was an infringement of its new broad product patent granted in Thailand. At the urging of civil society, GPO requested the Ministry of Public Health (MOPH) to grant a government use license to proceed with its production, but the request was rejected. Instead, the MOPH requested the GPO to produce the non-patented powder formulation of DDI, which was difficult to ingest and having gastrointestinal side-effects [27].

In 2001, the civil society coalition eventually challenged the BMS patent at the Thai Central Intellectual Property and International Trade Court on grounds that the patent on DDI included an unlawful amendment that effectively broadened the scope of the patent over all dosage strengths [28]. The amendment was ruled unlawful and the court also confirmed for the first time in Thailand, the right of individuals to challenge a patent [29], setting a major legal precedent in Thailand. This paved the way for the second challenge against the BMS patent, on grounds of lack of novelty (since the drug had already been on the market before it had been patented) and that the product patent application pre-dated the amended Patent Act [30]. A final ruling was never issued on this case since BMS settled the case by "dedicating" the patent to the Thai people in 2003 [31].

Since then, the civil society coalition has used legal challenges to defend and promote their right to access to medicines. The Health and Development Foundation filed a pre-grant opposition to the patent application from GlaxoSmithKline (GSK) on Combid/Combivir^®^, an ARV combination of lamivudine and zidovudine on grounds that the combination of two existing drugs was not an invention that merited patent protection [32]. Effective collaboration and sharing of information between civil society groups in India and Thailand allowed for the strategic filing of pre-grant oppositions simultaneously in Thailand and India. GSK subsequently withdrew this patent application in both Thailand and India in 2006 [33].

The passage of the National Health Security Act in 2002 resulted in the establishment of the Universal Healthcare Coverage (UC) scheme which ensures all Thai citizens the right to health care and access to medicines listed on the National List of Essential Medicines (NLEM). Further, the amended Thai Constitution of 2007 guarantees access to healthcare as a right, by explicitly stating that, *"(A) person shall enjoy an equal right to receive standard public health service, and the indigent shall have the right to receive free medical treatment from State's infirmary. The public health service by the State shall be provided thoroughly and efficiently. The State shall promptly prevent and eradicate harmful contagious diseases for the public without charge*".

Before 2003, the triple ARV drugs were not initially included in the NLEM due to the high prices of the drugs [34]. ARVs were only available for limited number of patients who had access to them under research or clinical projects or those who paid out-of-pocket. In October 2003, the government eventually declared its commitment to provide universal access to triple ARVs for HIV/AIDS treatment. This was due to a combination of sustained pressure from the movement of people living with HIV/AIDS (PLWHA) and civil society groups in Thailand, availability of low-cost triple therapy (GPO-VIR, produced locally by the GPO), and financial support from the Global Fund [35]. Although the government responded to this commitment by significantly increasing the national health budget, the budget increase was still not sufficient to meet the goal of universal access to ARVs in Thailand.

Significant financial resources were still required to ensure access to needed medicines, particularly in light of the need for second-line ARVs for patients who had developed drug resistance to first-line treatment. Moreover, there were also potential increased costs for the universal access scheme as changes were being considered for the first-line ARV treatment in Thailand. MOPH was considering the inclusion of efavirenz as a first-line ARV but had been unsuccessful in its negotiations for price reductions with the patent holder, Merck Sharp & Dohme (MSD), over the period between 2004-2005. Consequently, the MOPH initiated a feasibility study of the use of TRIPS flexibilities for efavirenz [22].

This section suggests that medicine patenting and its implications on access to medicines are not new issues in Thailand, but have been recognised and dealt with by various groups of government officials and health advocates. Confrontations between the Thai civil society coalition and the US government and pharmaceutical companies over such issues have taken place over two decades. It demonstrates how knowledge and evidence generated by researchers has been used by civil society for effective and strategic campaigns. This also shows that expertise and experience related to government use licenses have gradually accumulated prior to granting government use licences for the seven medicines in 2006 to 2008.

### Government and political support: completing the triangle

The major concern of the government in committing to universal access to essential health services under the UC scheme was the long-term sustainability of the government-funded health plan, particularly in light of the need for more expensive medicines (such as efavirenz and second-line ARVs). Increasing access to these patented ARVs was a central issue. The role of generic drugs in reducing the cost of HIV treatment has been well-illustrated by the introduction of much lower-priced Indian-made generic ARVs to the global market. Generic ARVs have revolutionised the scale-up of HIV treatment programmes in many developing countries, including Thailand. Increasing concerns about drug resistance to existing treatments, the prohibitive cost of second- and third-line ARV therapy and the patent barriers for local generic production were factors that played a significant role in convincing policy makers to seriously consider the use of TRIPS flexibilities, as a means to ensure universal access to ARVs.

Within the government, the National Health Security Office (NHSO) was mandated as the agency with responsibility for the payment, administration and management of the national program for universal access to HIV treatment. Accordingly, it was in the interest of the NHSO to consider the options for ensuring access to patented ARVs at affordable cost. As with civil society groups, the policy makers in NHSO also underwent a process of capacity building on intellectual property rights, pharmaceutical patents and the TRIPS Agreement - traditionally the domain of other government agencies - as the global debate on patents and public health unfolded. Participation at international fora, such as the World Health Assembly (WHA) and WTO meetings, as well as collaboration with international NGOs such as Knowledge Ecology Institute, Médecins Sans Frontières, Third World Network, and Oxfam contributed to their learning curve.

In early 2006, the need for an effective policy on these issues was discussed within the NHSO. The then National Health Securities Board appointed a "Subcommittee to Implement Government Use of Patented Medicines and Medical Devices", chaired by the Secretary General of the NHSO, to assess the need for the use of government use licenses and to develop the criteria to guide the selection of medicines for which the government use licenses was needed. Membership of the Subcommittee comprised senior officials from NHSO, MOPH, Food and Drug Administration (FDA), the Department of Intellectual Property, as well as representatives from health and consumer-protection groups in Thailand.

Efavirenz was initially identified as a candidate by the Subcommittee and a proposal to grant a government use licence for this medicine was submitted to the Health Minister [36]. The approval for the proposal, however, took considerable time and was also interrupted by the political turmoil in mid-2006 [37]. One of the reasons why the Health Minister at the time did not approve the grant of the government use licence was the fact that he was not familiar with the issue and had little background information on the impact of drug patents and TRIPS flexibilities [38]. The proposal thus remained with the MOPH legal office, pending further review [39]. A significant change took place when Dr. Mongkol Na Songkhla, the former permanent secretary of MOPH, was appointed Minister by the new military government in October 2006. The proposal from the Subcommittee was then seriously re-considered with support from civil society groups. This resulted in the decision to grant the government use licenses over the period 2006-2008.

Although the civil society coalition had enjoyed significant success in the legal challenges of DDI and combination of lamivudine and zidovudine patents, the government use licenses would have not been implemented effectively without strong support from politicians and the civil service technocrats. Many of the politicians and technocrats involved in the decision to grant the government use licenses shared a strong sense of public spirit that had been inculcated since they were medical students in mid 1970s^4^. The Health Minister also demonstrated both commitment to public health and leadership with clear directives, active involvement and unambiguous support for the government use licences. This was important in order to generate the required action from civil service technocrats [38].

In building the third point of the triangle, comprising the political and civil service sectors, it is not only the awareness and capacity among politicians and government officials to understand the information generated by scholars and civil society but also their commitment and leadership that were critical to the process. This final aspect completed the triangle, providing the political momentum to grant and implement the government use licences. Furthermore, it should also be noted that the successful implementation of the government use licences would not have been possible without a legitimate and transparent process for decision-making. The next section describes this process.

### Policy Implementation

In early 2007, two committees, namely the "Committee on Price Negotiation of Patented Essential Medicines" and the "Committee to Support Government-Use Implementation", were appointed by the Health Minister to facilitate the implementation of the government use licenses [40,41]. The establishment of these institutional mechanisms were aimed not only at ensuring access to affordable medicines, but also to respond to the concerns and critiques about the government use licenses.

The Committee on Price Negotiation of Patented Essential Medicines invited representatives of the relevant patent-holding pharmaceutical companies to discuss price reductions for the medicines under the government use licenses. It was believed that the announcement of government use licences would help to ensure the success of such price negotiations [42]. Although all of the pharmaceutical companies offered discounted prices and/or other programs to broaden access to medicines in the country [43], these did not comply with the benchmark set by the Health Minister - that the discounted prices should not be higher than 5% of the prices for the generic versions of the medicines. Imatinib was the only medicine for which the implementation of the government use licence was suspended because the company agreed to provide the original drug free to all patients with a household income of less than 1.7 million Baht per year [44]. This agreement would ensure that all the estimated 1,850 patients with chronic myeloid leukemia and gastrointestinal stromal tumor under the UC scheme have access to imatinib [45].

The Committee to Support Government-Use Implementation functioned as the coordinating forum for the related task forces under the MOPH and NHSO, and for the other government ministries, NGOs, academic institutes and individual experts. The Committee was also the focal point to address critiques and reactions from opponents of the government use licenses. The granting of these government use licenses had provoked a mixed reaction from governments, international organizations and civil society organizations. There was strong objection from the patent holding pharmaceutical companies. In the case of Abbott Laboratories, in March 2007 the company decided to withdraw its applications for marketing approval on seven new drugs in protest at the government use licenses on its product, Kaletra, the LPV/r combination. Subsequently, the Office of the United States Trade Representative (USTR), in its Special 301 Report of 2007, elevated Thailand from Watch List (WL) to Priority Watch List (PWL) on the grounds that "*in late 2006 and early 2007, there were further indications of a weakening respect for patents, as the Thai Government announced decisions to issue compulsory licenses for several patented pharmaceutical products. While the United States acknowledges a country's ability to issue such licenses in accordance with WTO rules, the lack of transparency and due process exhibited in Thailand represents a serious concern*". Further, on 1 July 2007, the USTR announced that privileges under the Generalized System of Preferences (GSP) were removed for three Thai products: gold accessories jewelry, polyethylene terephthalate, and flat screen television sets (U.S. Commercial Service, 2007).

In an effort to inform the public and to garner support, the Committee to Support Government-Use Implementation published two documents, the so-called "White Papers", which detail the rationale, legal issues and decision-making process of the government use licenses [44-46]. These White Papers were to improve public understanding of the rationale and the decision-making process for the government use licenses in order to help assure the legitimacy and validity of the decision. They were widely circulated both within and outside Thailand, and are also available on the MOPH's website. It can be said that the government has been successful in this respect and this also prevented succeeding Health Ministers from revoking the government use licenses. A further detailed study was conducted by an independent research arm of MOPH, the International Health Policy Program (IHPP), with the aim of documenting the policy processes involved in the decision to grant the government use licenses [39]. These documents are important not only for informing the public but also for clarifying the rationale behind the decisions on government use licenses.

In a similar move, the Health Minister who granted the government use licenses also sought support from WHO Member States at the WHA in 2007 by addressing the Assembly on these issues. The WHA that year adopted a resolution urging the WHO Director-General to provide technical and policy support to countries on the use of TRIPS flexibilities [47] and Thailand became the first country to request WHO support under this resolution. The mission, led by WHO with experts also from the WTO, UNDP and UNCTAD, produced a technical report in 2008 [48] that has been widely interpreted to confirm the validity of government use licenses and their compliance with the TRIPS Agreement.

## Conclusions

Having described the Thai experience with use of the TRIPS flexibility, specifically the government use licenses, the paper now concludes with some observations on lessons that may be useful for other countries. Firstly, implementing TRIPS flexibilities as a means to ensure access to medicines is a right that can be exercised by all WTO members. The Doha Declaration affirms that the TRIPS Agreement does contain a degree of flexibility that permits governments to consider different options when formulating laws and policy in relation to patent protection and public health. In Thailand, the granting of government use licenses was supported by the provisions in the Thai Patent Act B.E.2542 (A.D. 1999), which permit the use of compulsory licenses by the private and public sectors. Together with the National Health Security Act B.E.2545 (A.D. 2002), it enshrines the Thai people's right to health and universal access to essential health care and these legal provisions made a strong case for the government use of TRIPS flexibilities.

Secondly, the synergy between three sides of the triangle; a.) knowledge and evidence generation, b.) mobilization of civil society and public support, and c.) the leadership of politicians and policy makers, was key to the success in policy formulation and implementation. As already mentioned, research and technical capacity in the different but inter-related fields of intellectual law and public health is required to provide a solid evidence base for decision-making. This was illustrated by the Thai experience, where both civil society and the public sector were able to rely on information and evidence to support their case. The commitment to undertaking analysis and compiling information of policy to justify the decisions made were equally important. The granting of government use licenses in Thailand can be regarded as the result of years of learning and collaboration amongst scholars, the civil society coalition, and policy makers. Aside from the coordination and synergy of the three points of the triangles, windows of opportunity and timing can often be critical factors for positive outcomes. A number of developments in Thailand and on the international stage were instrumental in pushing forward the granting of government use licenses.

Thirdly, adequate management capacity at the national level and appropriate institutional mechanisms are vital for the implementation of TRIPS flexibilities. The establishment of an open and transparent process and the related committees, by which a collective decision could eventually be made within the Thai government, helped to ensure effective implementation of the government use licenses. Strategies to foster collaboration between the government authorities, civil society organizations, foreign experts, and international agencies in the relevant fields were also critical for mobilizing broad based support from other countries and actors.

Finally, there has been much criticism of the Thai government decision to grant government use licenses. While some critics challenged the legal validity of the licenses under international and domestic law, there are also those who question the political and economic costs of the grant of these licenses, in terms of trade sanctions imposed by foreign governments opposed to the government use licenses, and of pharmaceutical companies retaliating by withdrawing or delaying drug registrations in Thailand. These critics argue that these costs would far outweigh the benefits of the government use licenses. Another paper in this series will present the results of an attempt to assess the public health, economic and social impacts of the government use license with a view to clarifying aspects of the controversy and enabling a better informed, evidence-based debate between key stakeholders and offer further lessons learnt from Thailand's experience to decision makers in other settings.

## Competing interests

Non-financial competing interests

Suwit Wibulpolprasert was responsible for assessing the political risks and clarifying the legal aspects of the government use licenses. Vichai Chokevivat was the Chair of the Committee to Support Government-Use Implementation.

## Authors' contributions

SW and VC have made substantial contributions to conception and design. CO and IY have been involved in drafting and revising the manuscript. All authors have read and approved the final manuscript.

## Authors' Information

Dr Suwit Wibulpolprasert is the Senior Advisor on Disease Control and Prevention at the Ministry of Public Health. Dr Wibulpolprasert began his career as a general practitioner in the rural hospitals in Thailand, later becoming the Chairman of the Rural Doctors Society in 1984. He later became a Director of the Technical Division in the Food and Drug Administration of Thailand in 1991-1994. This was followed by a series of appointments to other senior positions within the Ministry of Public Health, including the Director of Bureau of Health Policy and Planning, Assistant Permanent Secretary, Deputy Permanent Secretary, and Senior Advisor at the Ministry of Public Health.

Dr Vichai Chokevivat is currently the chair of the Board of the Government Pharmaceutical Organisation of Thailand. He used to be the Chair of the Committee to Support the Implementation of the Government Use of Patents, established under the Ministry of Public Health. Dr Chokevivat used to be the Chair of the Rural Doctors Society in 1982 and received the Outstanding Rural Doctor Award from Siriraj Hospital and Medical School in 1986. He had also held the posts of Secretary-General of the Food and Drug Administration and Director-General of the Department for Development of Thai Traditional and Alternative Medicines, before retiring from his final post as Senior Advisor at the Ministry of Public Health in 2007.

## Endnotes

^1 ^The government use licence is a form of compulsory licensing, which permits the government to license the use of a patented invention to itself or a third party, without consent of the patent holder. Such use of a patent is permitted under the provisions of Article 31 of the TRIPS Agreement, which allows for the "public non-commercial use" of a patent by a government without the authorization of the patent holder.

^2 ^The 1997 Constitution, known as the "People's Constitution", is a landmark in democratic constitutional reform. It provides the basis for the protection of constitutional rights and civil liberties of the Thai people. The drafting and promulgation of the Constitution illustrates the successful application of "the triangle that moves the mountain". The first point of triangle was the Committee for Democratic Development, which was responsible for generating the relevant knowledge and evidence to formulate recommendations for political reforms. The other points of the triangle were represented by the public and civil society organizations, which mobilized public support for the reforms and the political actors and the bureaucracy, which supported the process of political reform. The convergence of the three points of the triangle provided the momentum needed to move forward the process of political reform that led to the People's Constitution.

^3 ^Drug Study Group is a civil society coalition in Thailand set up in 1975. The group consists of academia from various universities who are working on the issues of access to essential drugs, rational drug use and consumer protection.

^4 ^These politicians and technocrats were former leaders of the Rural Doctor Society, a strong civil society organization. It was set up by these leaders since 1978 and is still an influential organization with high social credibility.
